# Optimizing the Tensile Strength of Weld Lines in Glass Fiber Composite Injection Molding

**DOI:** 10.3390/ma17143428

**Published:** 2024-07-11

**Authors:** Tran Minh The Uyen, Hong Trong Nguyen, Van-Thuc Nguyen, Pham Son Minh, Thanh Trung Do, Van Thanh Tien Nguyen

**Affiliations:** 1 Faculty of Mechanical Engineering, Ho Chi Minh City University of Technology and Education, Ho Chi Minh City 71307, Vietnam; uyentmt@hcmute.edu.vn (T.M.T.U.); nvthuc@hcmute.edu.vn (V.-T.N.); minhps@hcmute.edu.vn (P.S.M.);; 2 Faculty of Mechanical Engineering, Industrial University of Ho Chi Minh City, Nguyen Van Bao Street, Ward 4, Go Vap District, Ho Chi Minh City 70000, Vietnam

**Keywords:** weld line, PA6 30% GF, tensile strength, injection molding, optimization design

## Abstract

Weld line defects, commonly occurring during the plastic product manufacturing process, are caused by the merging of two opposing streams of molten plastic. The presence of weld lines harms the product’s aesthetic appeal and durability. This study uses artificial neural networks to forecast the ultimate tensile strength of a PA6 composite incorporating 30% glass fibers (GFs). Data were collected from tensile strength tests and the technical parameters of injection molding. The packing pressure factor is the one that significantly affects the tensile strength value. The melt temperature has a significant impact on the product’s strength as well. In contrast, the filling time factor has less impact than other factors. According to the scanning electron microscope result, the smooth fracture surface indicates the weld line area’s high brittleness. Fiber bridging across the weld line area is evident in numerous fractured GF pieces on the fracture surface, which enhances this area. Tensile strength values vary based on the injection parameters, from 65.51 MPa to 73.19 MPa. In addition, the experimental data comprise the outcomes of the artificial neural networks (ANNs), with the maximum relative variation being only 4.63%. The results could improve the PA6 reinforced with 30% GF injection molding procedure with weld lines. In further research, mold temperature improvement should be considered an exemplary method for enhancing the weld line strength.

## 1. Introduction

**Figure 1:**
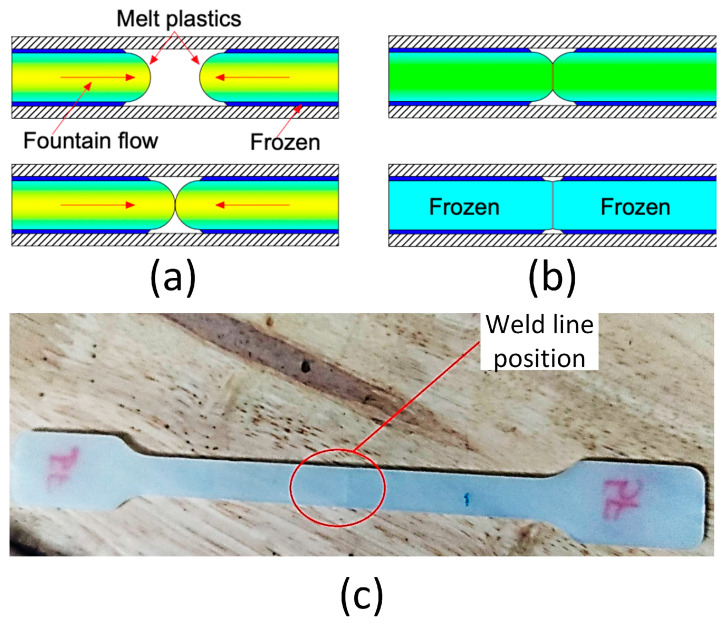
The formation process of weld lines in injection molding: (**a**,**b**) the weld line formation process, as well as (**c**) the weld line position in the sample.

Injection molding is a critical method in the plastics industry [1,2,3]. During the injection molding, the plastic gradually solidifies as it cools and transfers heat to the mold. Figure 1 depicts how a weld line emerges when two molten plastic flows interact in the opposite direction during the injection molding process. A weld line is formed in four stages: cooling, merging, commencement, and complete development [4,5,6,7]. Weld lines in injection-molded products might reduce the part’s strength and lifespan [8,9]. It is a weak area, lowering the injection product’s overall durability and strength. Manufacturers can improve the quality of injection products by limiting the negative impacts of this weakness type.

Many authors have tried to improve the weld lines’ strength. For example, Liparoti et al. [10] investigated the weld lines’ strength in micro-injection molding. They surveyed the influences of mold temperature on the weld lines’ characteristics, indicating that the weld lines’ strength could be enhanced when the mold temperature obtains at least 100 °C.

Purgleitner et al. [11] examined the effects of materials’ characteristics and the injection process on the weld line properties. They concluded that increasing the mold temperature and melt flow rate has a more substantial impact than changing other parameters. Kitayama et al. [12] optimized the injection molding process to improve the weld lines strength. They indicated that a higher mold temperature and a shorter injection time led to a higher weld line strength. Scantamburlo et al. [13] examined the impact of the PP polymer reinforced with GF inflow on its weld line strength. They proved that controlling the inflow could improve the strength of 19% of the weld lines. Liu et al. [14] simulated the weld lines’ location and characteristics, revealing that the weld lines could be predicted accurately. Hassan et al. [15] investigated the tensile strength, impact strength, and fiber length properties of the PA 6, 6 composites reinforced with GF. The study indicated that the tensile strength, tensile modulus, and impact strength are improved when the fiber volume is increased. Moreover, with increased fiber volume fraction, more fiber degradation occurred through the composite material processing. Lionetto et al. [16] examined the relationship between the elastic characteristics and morphology of the short fiber composites via X-ray tomography. The results show that most fibers are aligned in the injection direction, as the fabric tensor indicates. Interestingly, a micro/macro mechanical model for determining the elastic modulus of unidirectional short-fiber composites has been successfully proposed based on a correlation between the morphological results and the elastic characteristics of the sample.

**Figure 2:**
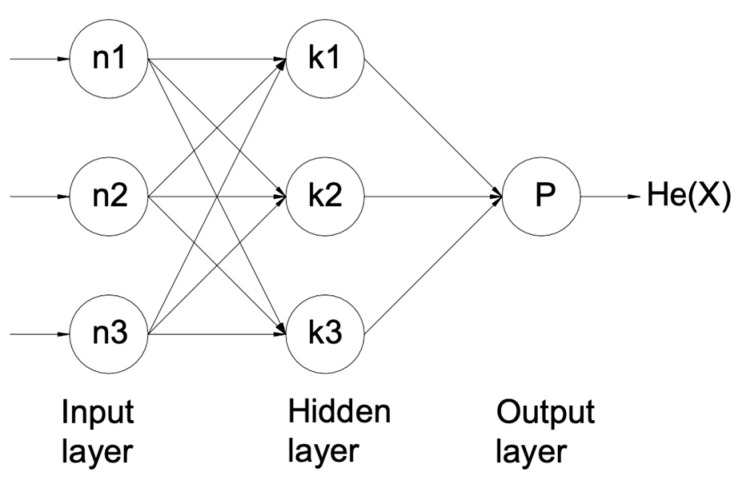
Artificial neural network structure.

Artificial neural networks (ANNs) are computational algorithms inspired by biological systems that aim to accurately indicate the link between n-dimensional input and output vectors [17,18]. An ANN comprises two layers: an input layer with n nodes providing n input variables and an output layer with p nodes reflecting p output [19,20,21]. The hidden layers sit between the input and output layers. Each hidden layer has k-hidden neurons, with the value of k determined subjectively. Figure 2 illustrates the links between these three layers. The network designer initially determined the weights assigned to these connections among the three layers, but they are subsequently modified for every “epoch” that the network goes through. Shen et al. [22] optimized the injection molding parameter using ANN and genetic algorithm methods. The results reveal that the ANN methodology could effectively model the complex interaction between process conditions and quality index for injection molded parts. Shi et al. [23] also focused on optimizing the injection molding parameters of a cellular phone cover by using ANNs and the expected improvement function method. The study shows that the ANN method might decrease warpage in injection molded parts and may quickly converge to the optimal solution. Although the design variables are only confined to mold temperature, melt temperature, injection time, packing pressure, packing time, and cooling time, this method can be applied to a broader range of process parameters. Lee et al. [24] examined the accuracy of ANN prediction in the injection molding process by considering the effects of input parameter range. Input parameters included melt temperature, mold temperature, injection speed, packing pressure, packing time, and cooling time. The injection-molded product’s mass, diameter, and height are chosen as output parameters for building an ANN mode. The performance of ANN prediction was compared to that of linear and second-order polynomial regressions. In the datasets, the anticipated results of ANNs outperformed those of linear regression and second-order polynomials. In addition, ANNs have numerous advantages, including storing information across the entire network, functioning with partial knowledge, being fault-tolerant, and processing data in parallel. However, some disadvantages of ANNs include hardware reliance, inexplicable behavior, difficulties establishing network structure, and the unknown ideal training duration [25].

Surveying multi-parameters and applying ANNs to predict the weld lines’ strength is necessary to improve the injection products. However, it is rarely mentioned. This research employs ANNs to explore the ultimate tensile strength of a PA6 material with 30% glass fiber (GF). The impacts of filling, packing parameters, and the temperature of molten plastics are investigated. After injection, the samples undergo a tensile test. After that, the experiment results are processed via the ANN tool. The results could help optimize the injection molding process of PA6 30% GF with weld lines.

## 2. Experimental Methods

**Figure 3:**
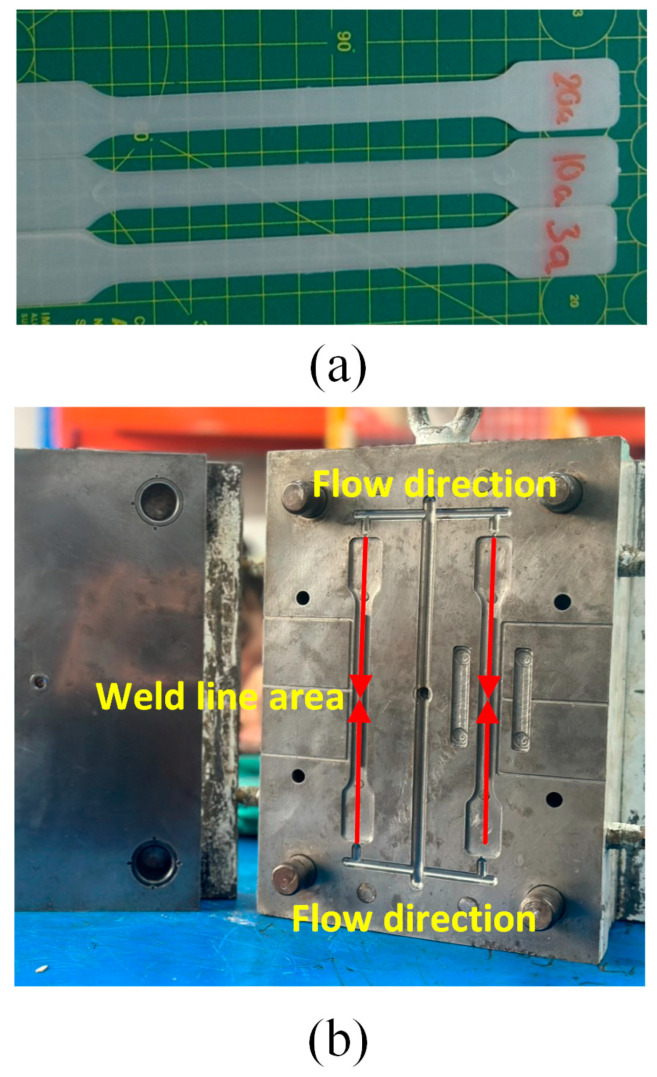
PA6 with 30% glass fiber samples after injection and the mold shape in the opposite direction between melt flow: (**a**) Sample shape corresponding to the ASTM D638 standards, (**b**) The mold with the core and cavity plates.

**Figure 4:**
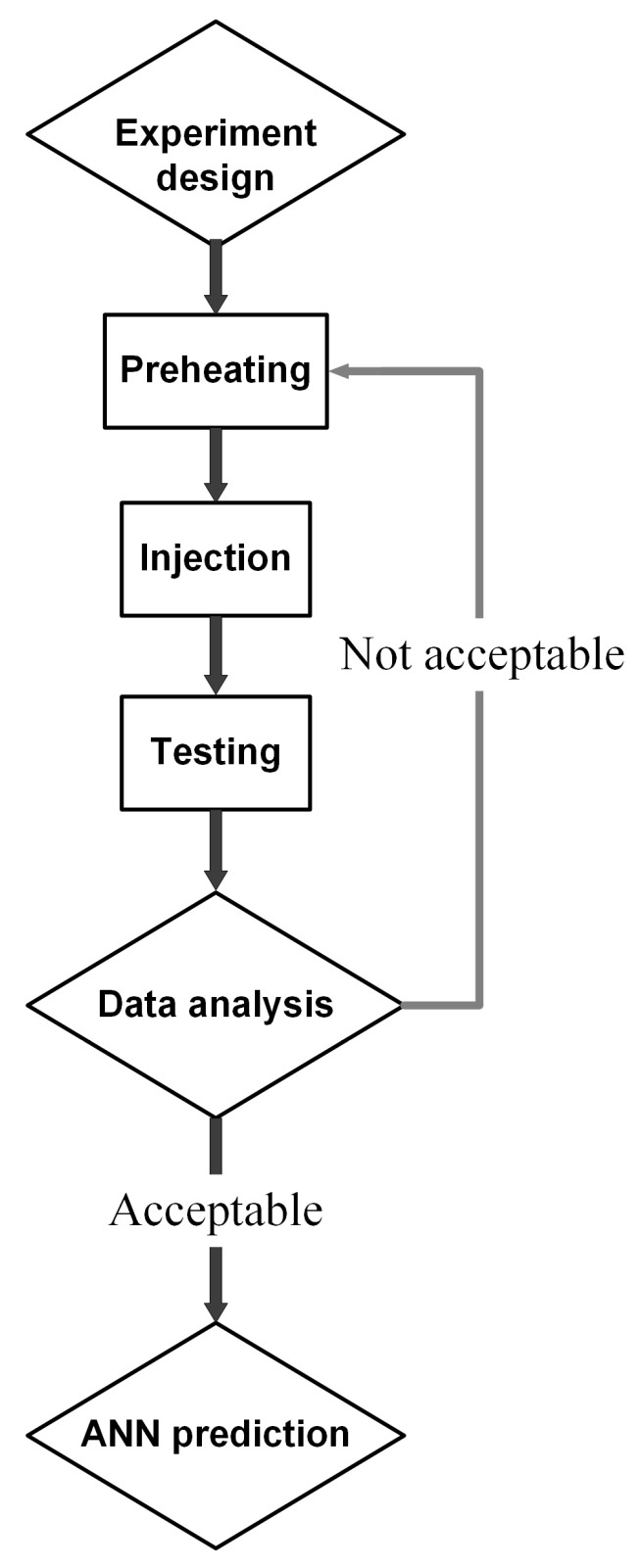
The investigation procedure of the weld line strength of PA6 reinforced with 30% glass fibers.

Figure 3a displays the sample shape corresponding to the ASTM D638 standards [26]. The mold is designed with the core and cavity plates, as shown in Figure 3b, to ensure the formation of the weld line. The “weld line areas” are indicated on both the traditional and innovative cavities, demonstrating the precise sites where weld lines generally develop. Weld lines are locations where two flow fronts come together during the molding process and might be weak points in the completed product. Finally, the venting gaps are indicated on both sides of the cavity plate. These spaces are critical for enabling air to escape from the mold as the material is injected, preventing flaws in the finished product due to trapped air. Figure 4 outlines the experimental methodology. Initially, the PA6 plastic containing 30% GFs, supplied from Akulon^®^ K224-G6 by DSM Company (Heerlen, The Netherlands), is heated at 80 °C for 8 h to eliminate moisture. This plastic obtains an ultimate tensile strength (UTS) of 110 MPa, a 7% elongation at break, and a melting temperature range from 270 °C to 290 °C. The chemical composition and typical properties of this PA6 plastic is presented in Table 1. The injection molding equipment is the MA 1200II model from Haiti, China. In addition, before the experiments following Table 2, the sample injection is conducted several times to avoid injection defects such as sink marks, short shots, or wrapping. Each sample number has five samples for the test. The tensile test values are presented as average figures with error bars to indicate the mechanical property deviations from the mean.

In this study, we did not investigate the effect of the mold temperature on the weld line formation. Increasing the mold temperature could enhance the weld line strength. However, more components must be added to the mold, making it more complicated. During the experiment, the mold opens and closes repeatedly; it is cooled down by the outside environment, leading to a temperature of around 30 °C. This issue could be an interesting topic that will be investigated further.

The control over pressure, temperature, and other fill parameters is achieved through a Haitian Techmation Tech2 controller (Haitian, Ningbo, China), which includes a control panel. These parameters can be adjusted using this control panel interface. To examine injection parameters, the study looks into the packing time, packing pressure, filling time, filling pressure, and melt temperature, as detailed in Table 2. Packing time is when pressure is applied to the molten plastic material inside the mold. Packing time aims to adjust for material shrinkage as it cools and solidifies, ensuring that the finished molded item has the correct dimensions, strength, and surface polish. Packing pressure is imposed by densely packed molten plastic molecules inside an injection barrel. If packing pressure is insufficient, cavities and air spaces occur in a material. As a result, it is critical to maintain adequate packing pressure for an extended period.

In the first group comprising samples between No. 1 and No. 5, they were filled at a range between 3.0 and 3.8 s, with a constant filling pressure of 64 MPa, a packing time of 0.4 s, a packing pressure of 59 MPa, and a melt temperature of 269 °C to study the influence of filling time on sample characteristics. Then, the following group, with samples from No. 6 to No. 10, explores the filling pressure effects within a 60–68 MPa range. The next group, with samples from No. 11 to No. 15, explores the packing time effects within a 0–0.8 s range. Group four, containing samples from No. 16 to No. 20, examines the impact of varying packing pressures between 55 and 63 MPa. The fifth group, with samples from No. 20 to No. 25, surveys how altering the melt temperature within a 265–273 °C range affects sample properties. The injection samples are examined using an ASTM D638 standard with the tensile test equipment AG-X Plus 20 kN (Shimadzu, Kyoto, Japan). After that, the fracture surfaces are studied using a scanning electron microscope (SEM) TM4000 (Hitachi, Ibaraki, Japan).

By ASTM D638 guidelines [26], the injection samples are tested using a Shimadzu AG-X Plus 20 kN tensile test apparatus (Shimadzu, Kyoto, Japan) at a 5 mm·min^−1^ speed and a grasp spacing of 135 mm.

## 3. Results and Discussion

Initially, the study explored the impacts of varying the injection molding filling time, which ranges from 3.0 s to 3.8 s.

**Figure 5:**
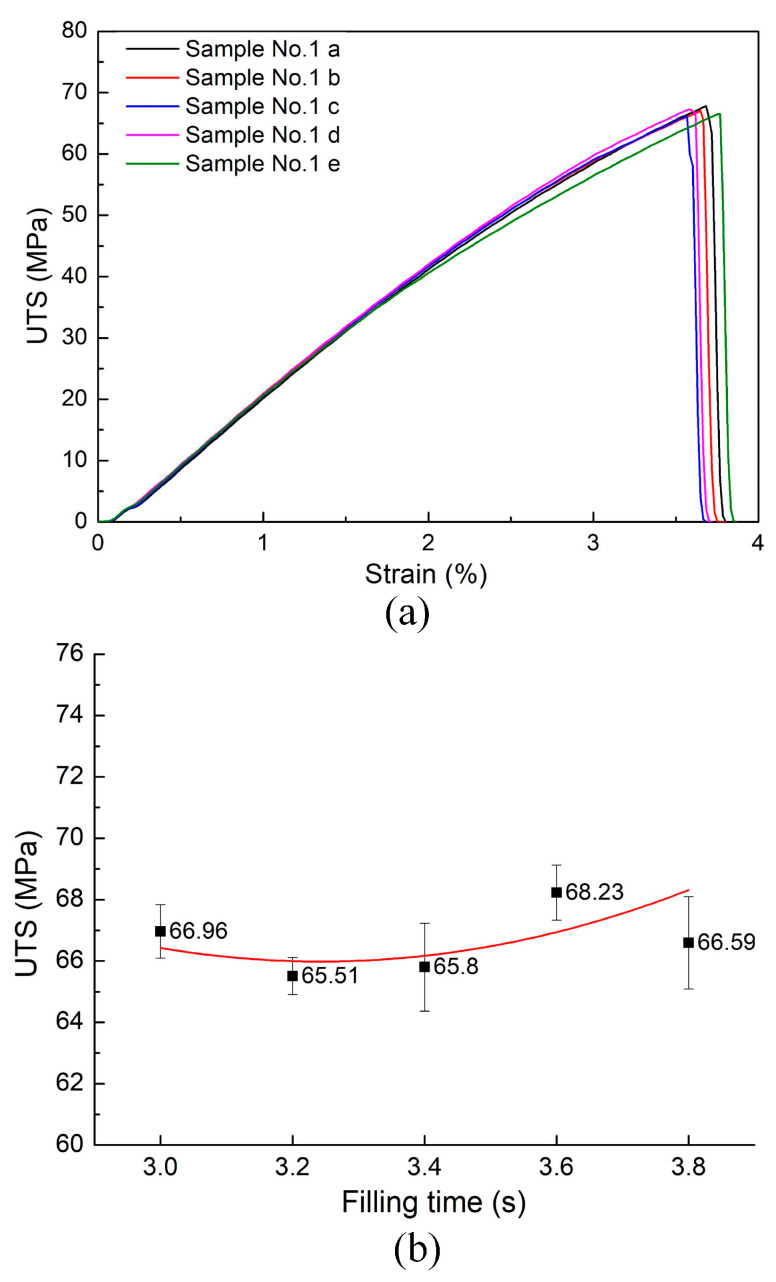
Stress–strain curve of PA6 reinforced with 30% GFs and average UTS values of PA6 reinforced with 30% GF composite samples with weld lines at various filling times: (**a**) stress–strain curve of samples No.1a–e, and (**b**) average UTS comparison.

Figure 5a presents the stress–strain curve for a PA6 reinforced with 30% GF composite samples with weld lines, revealing that the sample elongation is below 5%. This indicates the brittle characteristic in the weld line area due to poor bonding properties. Figure 5b compares the composite samples’ ultimate tensile strength (UTS) with weld lines across various filling times. The filling time parameter range is 3.0–3.8 s. The average UTS is calculated to be 66.02 MPa, with a deviation from the mean of 1.07 MPa. This tensile strength value is lower than that of the original composite samples without a weld line, which is 110 MPa, demonstrating the detrimental influence of the weld line on the bonding quality. The weld line formation interrupts the continuity of the polymer network of the injected samples, reducing its tensile strength. Moreover, the injection process could also cause a fiber orientation disorder, reducing the weld line strength [15]. The greatest UTS observed was 68.23 MPa at 3.6 s, while the lowest was 65.51 MPa at 3.2 s, indicating a relatively slight change in UTS across samples and an existence minimal value of the UTS. The filling periods of 3.0 to 3.8 s are regarded as appropriate for the injection procedure, suggesting that the UTS values fluctuate slightly, from 65.51 MPa to 68.23 MPa, within this range.

**Figure 6:**
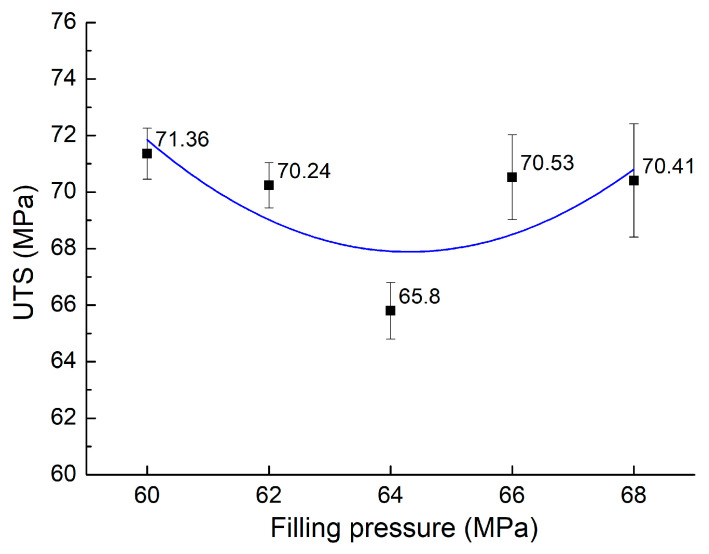
Average UTS values of PA6 reinforced with 30% GF composite samples with weld lines at various filling pressures.

The UTS values of composite samples made of PA6 reinforced with 30% GFs and the weld lines at different filling pressures from 60 MPa to 68 MP are displayed in Figure 6. The average UTS calculated across all conditions is 69.67 MPa, with a standard deviation of 2.20 MPa. The standard deviation value when changing the filling pressure is higher than when changing the filling time. This indicates that the UTS value is more sensitive to the filling pressure than the filling time when surveying these parameters in the mentioned ranges. This result is similar to Raos et al.’s report [34], which studies the polyethylene material’s tensile strength, indicating the filling pressure’s high influence compared to the filling time (or injection speed). The highest UTS value of 71.36 MPa is obtained at 60 MPa, the lowest value of 65.8 MPa is achieved at 60 MPa, and the lowest value is 65.8 MPa when pressing at 64 MPa.

**Figure 7:**
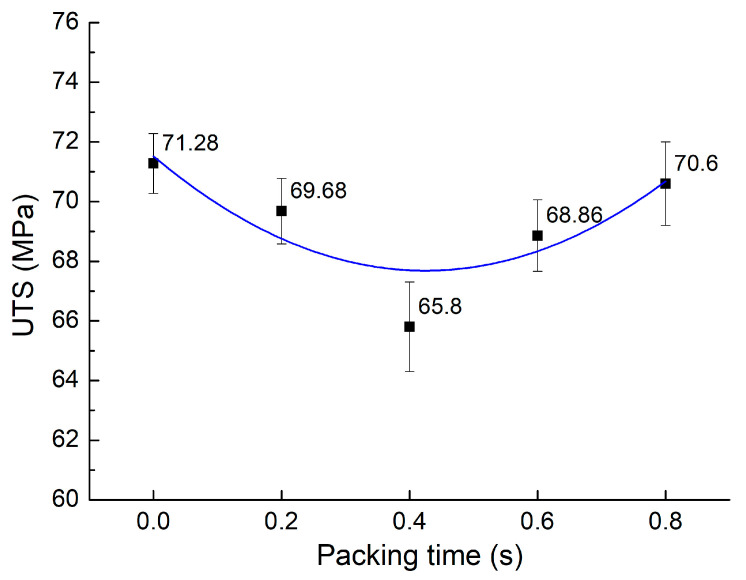
Comparison of average UTS values of PA6 reinforced with 30% GF composite samples with weld lines at various packing times.

Packing time is the extra phase of applying pressure after injecting the molten plastics into the mold. Applying the packing phase can eliminate the presence of air bubbles within the injected sample, hence improving the injection-molded product’s overall quality. The air bubbles could appear due to residual humidity in the plastic granulation, the absorption of air in the atmosphere, and the air inside the mold. Applying a packing period could eliminate the presence of air bubbles. This research examines the impact of packing times ranging from zero to 0.8 s. Figure 7 displays the UTS values for PA6 reinforced with 30% GF composite samples with weld lines at various packing times. The mean UTS across all these conditions is calculated to be 69.24 MPa, with a standard deviation of 2.13 MPa. The results show that the impact of applying the packing phase is not as strong as the presence of GFs. The GF appears to be the linking bridge in the weld line area, increasing the weld lines’ strength. Overall, with the PA6 reinforced with 30% GF composite, the packing time is less critical than the polymer without GFs. According to Singh et al.’s report [35], introducing the packing time could cause a minor reduction in the tensile strength. Therefore, the packing time needs more investigation in future work by increasing it with the broader range.

**Figure 8:**
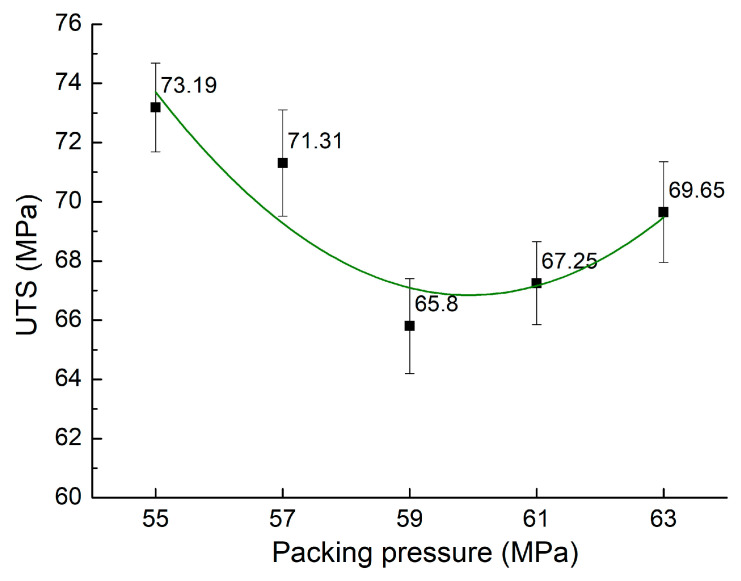
Comparison of UTS values of PA6 reinforced with 30% GF composite samples with weld lines at various packing pressures.

In addition to packing duration, the packing pressure factor may also strongly impact the strength of the weld lines. Shokri et al. [36] suggested that control of the packing pressure could change the orientation of the fiber; therefore, it could strongly impact the tensile strength of the reinforced polymer. Figure 8 illustrates the tensile strength for PA6 reinforced with 30% GF composite samples with weld lines under various packing pressures of 55–63 MPa. The average UTS across these conditions is 69.44 MPa, with a standard deviation of 2.99 MPa, indicating that packing pressure variations have a more pronounced impact on UTS than variations in packing time, filling pressure, and filling time, as evidenced by the higher standard deviation. Moreover, the average UTS value associated with packing pressure, 70.94 MPa, is slightly higher by 0.8 MPa than observed in the packing time scenarios. The PA6 reinforced with 30% GF composite sample under 55 MPa packing pressure had the maximum UTS of 73.19 MPa among the tested conditions, while its lowest UTS of 65.80 MPa was identified at 59 MPa. Comparable to the packing time scenarios, the packing pressure of PA6 reinforced with 30% GF composites is less critical than that of polymer without GFs.

**Figure 9:**
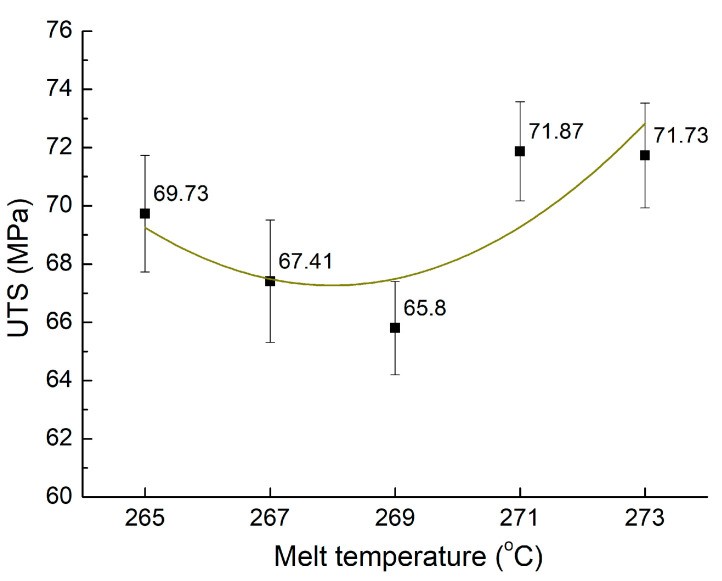
Comparison of UTS values of PA6 reinforced with 30% GF composite samples with weld lines at various melt temperatures.

The melt temperature of the plastics significantly influences the injection molding process. The viscosity of the material could rise at a low melt temperature, hindering the filling process. On the other hand, melt temperatures that are too high may cause polymer degradation. This study looks at how the melt temperature affects the mechanical characteristics of PA6 reinforced with 30% GF composite samples. The range of temperatures is 265 °C to 273 °C. The UTS of PA6 reinforced with 30% GF composite samples with weld lines at different melt temperatures is displayed in Figure 9. Under these conditions, the UTS averages 69.31 MPa, with a standard deviation of 2.67 MPa.

The peak UTS of 71.87 MPa is achieved at a melt temperature of 271 °C, whereas the lowest UTS of 65.8 MPa is observed at 269 °C. Furthermore, samples processed at higher melt temperatures of 271 °C and 273 °C exhibit greater UTS values compared to those processed at the lower temperatures of 265 °C, 267 °C, and 269 °C. This trend suggests that the higher temperatures facilitate a smoother flow rate, leading to improved weld line quality, consistent with Kitayama et al.’s report [12]. In addition, thin-wall injection molding productions are more sensitive to the weld line as the mold could rapidly absorb the heat and the melt polymer could be solidified [37]. The weld line negative effect, therefore, could be increased. Increasing the melt temperature is a good method to improve the weld line strength. Therefore, applying this result to the thin-wall product could enhance its stability.

**Figure 10:**
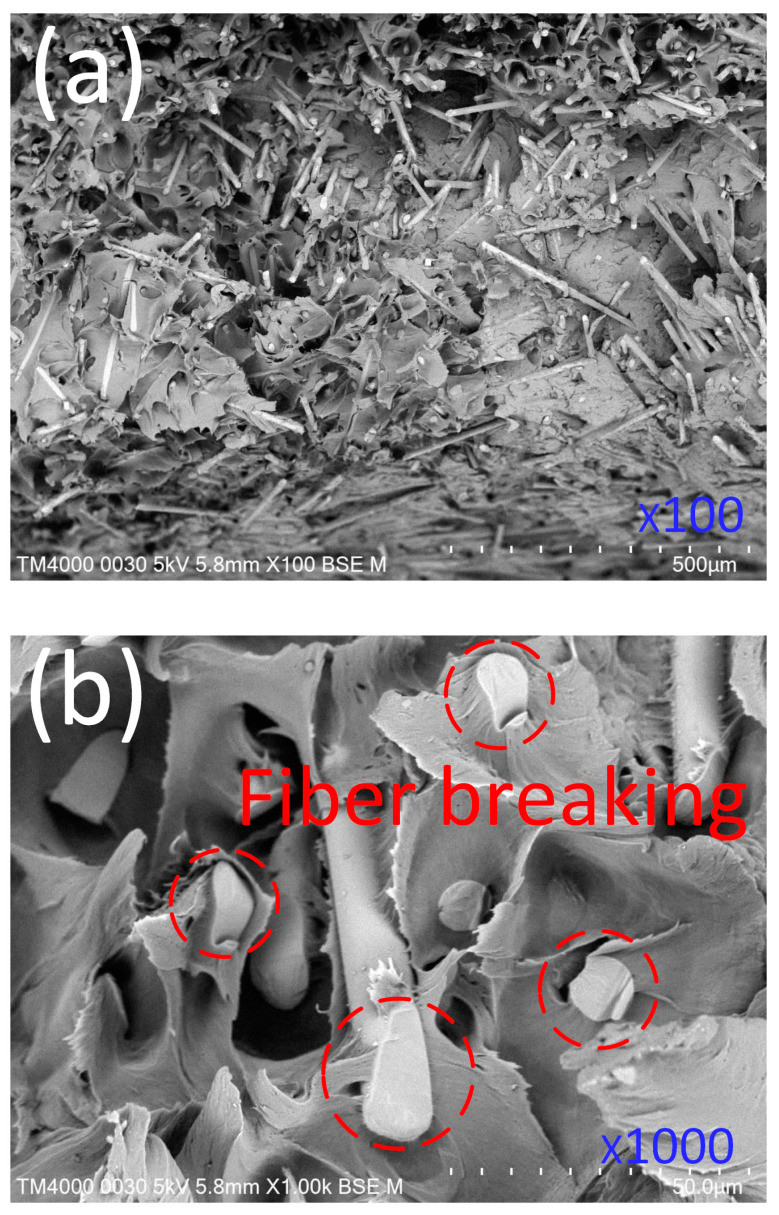
The fracture surface of PA6 was reinforced with 30% GFs with a weld line under a SEM. (**a**) ×100 magnification, (**b**) ×1000 magnification.

Figure 10 displays the fracture surfaces of the PA6 reinforced with a 30% GF composite sample via SEM. The PA matrix has a dispersion of GFs. There appears to be a strong bond between the GFs and the PA matrix at their boundary. Cracks occur at the ends of the fibers during the tensile process. Cracks propagated along the interface through the matrix. Finally, matrix fractures are caused by interfacial cracks, which could result from matrix plastic deformation. The smooth fracture surface indicates the high brittleness of the PA6 reinforced with a 30% GF sample. Low elongation values also represent brittleness in the weld line area, resulting in fiber breaking, as shown in Figure 10b. Moreover, high shear stress during the injection molding could also cause fiber breaking [16]. Notably, under high shear stress of the injection pressure, the fiber orientation is aligned in the injection direction or perpendicular to the weld line area. Many GF fragments on the fracture surface have cracked, showing fiber bridging across the weld line area. This fiber bridging mechanism is the reason for the increase in the weld line’s strength [38].

Previous results have primarily focused on optimizing a single parameter. This section expands the scope to include a broader view by comparing the standard deviation of the UTS value. Table 3 displays the average UTS and standard deviations for PA6 reinforced with 30% GF composite samples featuring weld lines subjected to various injection factors. The standard deviation is 1.07, 2.20, 2.13, 2.99, and 2.67, corresponding to filling, filling, packing, packing, and melting temperature. The filling time factor’s standard deviation for the UTS is 1.07, the smallest recorded, indicating its minimal impact compared to other factors. Conversely, the packing pressure factor shows the most excellent standard deviation of 2.99, highlighting its significant influence on the UTS value. The melt temperature plays a crucial effect on the strength of the product, as seen by its high standard deviation value of 2.67. The regression equation created by Minitab 20.4 software also indicated the effects of these parameters:

UTS = 0.99 × Filling time − 0.080 × Filling pressure − 1.09 × Packing time − 0.557 × Packing pressure + 0.423 × Temperature − 10(1)

This equation shows that increasing the values of filling time and temperature leads to an increase in the UTS value. In contrast, increasing the values of filling pressure, packing time, and packing pressure results in a decrease in the UTS value. However, increasing the filling time and temperature could only benefit within a suitable range. Too much filling time could reduce filling pressure, while the polymer may degrade if the melting temperature is too high.

**Figure 11:**
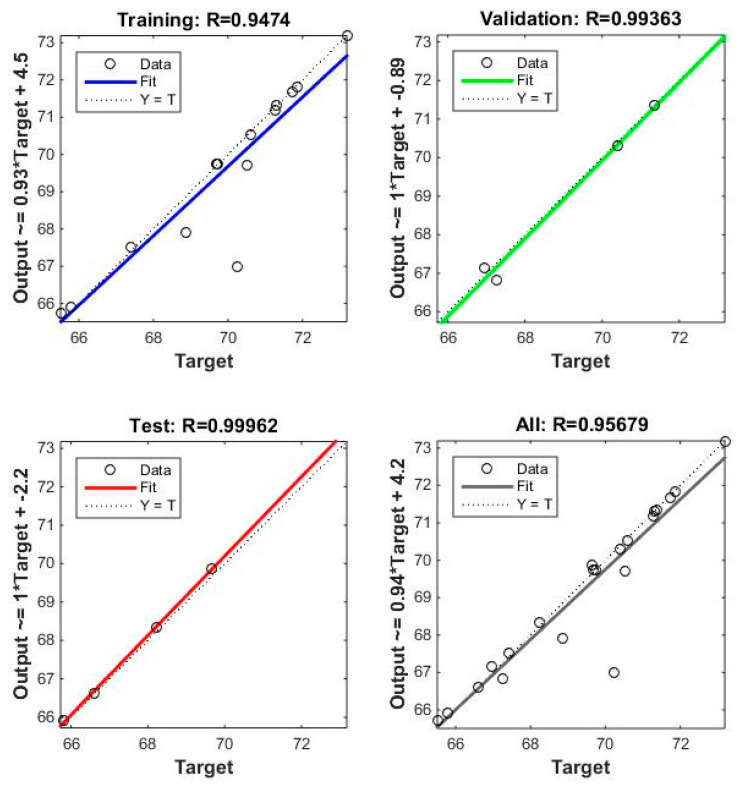
The PA6 reinforced with 30% GF composite samples with weld lines’ R-squared results.

**Figure 12:**
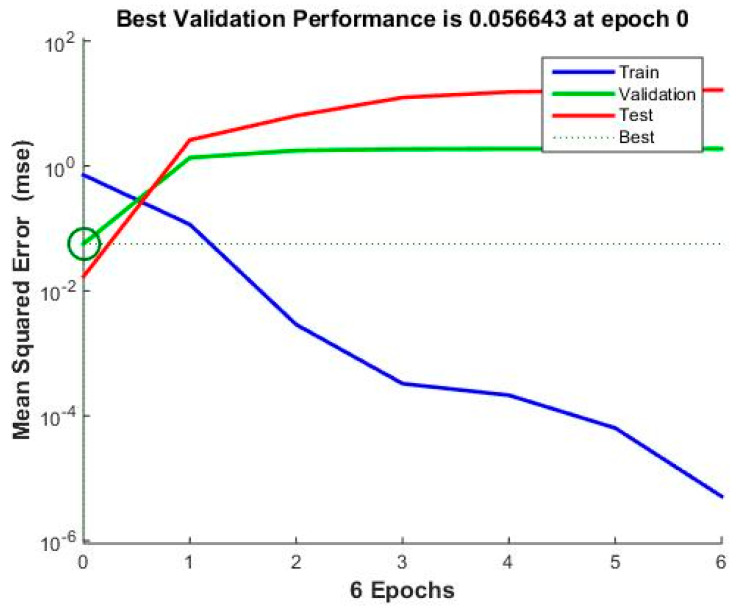
The PA6 reinforced with 30% GF composite samples with weld lines’ validation performance.

Figure 11 shows that the neural network model has a solid and consistent relationship between the outputs and the target values across all datasets (training, validation, and test). The R values are close to 1, indicating the consistency of the network output and experiment results, as presented in Table 4. Figure 12 shows that the training process has significantly improved performance on the training set, with MSE decreasing sharply over the epochs. However, the performance on the validation and test sets stabilizes after a few epochs, with the best performance achieved very early in the training process.

Apart from illustrating the importance of each parameter in Table 3, we used an optimization technique called ANNs. Instead of using explicit programming, an ANN learns through experience. It is designed to identify patterns and correlations in the data from Table 2. We used MATLAB R2014a to create a feed-forward backpropagation neural network with the Levenberg–Marquardt training function, consisting of 25 hidden neurons using the Tansig activation function, one output node, and five input nodes. The network’s reliability was evaluated by examining the R-squared values and Mean Squared Error (MSE). Table 4 displays the experimental testing results and network output for PA6 reinforced with 30% GFs. It can be demonstrated that, with the most significant relative variation being only 4.63%, the expected outcomes of the ANN essentially correspond with the experimental data.

The parameter range in this study still has a limitation value. To improve the application ability, in future research, the study should investigate a wider range, with many types of polymer and using some advanced techniques such as a heat-assisted mold system, adding more additives. The other types of properties such as bending strength, ductility, and impact strength also need more investigation.

## 4. Conclusions

In this study, we employ artificial neural networks to explore the ultimate tensile strength of a PA6 composite with 30% glass fibers. The impacts of filling time, filling pressure, packing time, packing pressure, and melt temperature on the UTS value are investigated. The factor with the most significant influence on the UTS value is the packing pressure component. The strength of the product is also significantly influenced by the melt temperature. Conversely, the filling time factor has less effect on other factors. The SEM result shows that the smooth fracture surface indicates the high brittleness in the weld line area. Many GF fragments on the fracture surface have cracked, showing fiber bridging across the weld line area, enhancing this area. UTS values range from 65.51 MPa to 73.19 MPa, depending on the injection parameters. Additionally, the experimental data often comprise the outcomes of the ANN, with the maximum relative variation being only 4.63%. The results may enhance the PA6 reinforced with 30% GF injection molding procedure with weld lines. In further investigation, the mold temperature should be increased as it is an excellent technique to improve the weld line strength. Boarder parameter range, polymer type, additives, and other mechanical properties also need more consideration.

## Figures and Tables

**Table 1:** Chemical composition and typical properties of PA6 reinforced with 30% GFs from Akulon^®^ K224-G6 by DSM.

Properties	Typical Data	Unit	Test Method
Chemical composite	70% Polyamide 6 ((C_6_H_11_NO)_n_) + 30% glass fibers	-	-
Molding shrinkage, parallel	0.3	%	ISO 294-4, 2577 [27]
Molding shrinkage, normal	0.9	%	ISO 294-4, 2577
Tensile modulus	9700/6000	MPa	ISO 527-1/-2 [28]
Stress at break	185/110	MPa	ISO 527-1/-2
Strain at break	3.8/7	%	ISO 527-1/-2
Charpy impact strength, +23 °C	95/110	kJ·m^−2^	ISO 179/1eU [29]
Shore D hardness	-/85	-	ISO 48-4 [30]
Melting temperature, 10 °C/min	220	°C	ISO 11357-1/-3 [31]
Water absorption	6.3	%	Sim. to ISO 62 [32]
Humidity absorption	1.9	%	Sim. to ISO 62
Density	1350/-	Kg·m^−3^	ISO 1183 [33]
Thermal conductivity of melt	0.27	W·(m K)^−1^	-

**Table 2:** Injection molding parameters and average UTS values of samples with PA6 reinforced with 30% GFs.

No.	Filling Time (s)	Filling Pressure (MPa)	Packing Time (s)	Packing Pressure (MPa)	Melt Temperature (°C)	UTS (MPa)
1	3					66.96
2	3.2					65.51
3	3.4	64	0.4	59	269	65.8
4	3.6					68.23
5	3.8					66.59
6		60				71.36
7		62				70.24
8	3.4	64	0.4	59	269	65.8
9		66				70.53
10		68				70.41
11			0			71.28
12			0.2			69.68
13	3.4	64	0.4	59	269	65.8
14			0.6			68.86
15			0.8			70.6
16				55		73.19
17				57		71.31
18	3.4	64	0.4	59	269	65.8
19				61		67.25
20				63		69.65
21					265	69.73
22					267	67.41
23	3.4	64	0.4	59	269	65.8
24					271	71.87
25					273	71.73

**Table 3:** Average UTS values and standard deviations for PA6 reinforced with 30% GF composite samples with weld lines at various injection factors.

Factors	Average UTS (MPa)	Standard Deviation (MPa)
Filling time	66.02	1.07
Filling pressure	69.67	2.20
Packing time	69.24	2.13
Packing pressure	69.44	2.99
Melt temperature	69.31	2.67

**Table 4:** A comparison of the experimental testing data for PA6 reinforced with 30% GFs with the network output.

No.	Experiment (MPa)	Network Output (MPa)	Relative Deviation (%)
1	66.96	67.15	0.28
2	65.51	65.72	0.32
3	65.80	65.90	0.15
4	68.23	68.33	0.15
5	66.59	66.62	0.05
6	71.36	71.36	0.00
7	70.24	66.99	4.63
8	65.80	65.90	0.15
9	70.53	69.72	1.15
10	70.41	70.29	0.17
11	71.28	71.19	0.13
12	69.68	69.74	0.09
13	65.80	65.90	0.15
14	68.86	67.89	1.41
15	70.60	70.52	0.11
16	73.19	72.17	1.39
17	71.31	71.32	0.01
18	65.80	65.90	0.15
19	67.25	66.83	0.62
20	69.65	69.86	0.30
21	69.73	69.73	0.00
22	67.41	67.51	0.15
23	65.80	65.90	0.15
24	71.87	71.82	0.07
25	71.73	71.68	0.07

## Data Availability

The raw data supporting the conclusions of this article will be made available by the authors on request.
